# Spontaneous Self-Assembly of Single-Chain Amphiphilic Polymeric Nanoparticles in Water

**DOI:** 10.3390/nano10102006

**Published:** 2020-10-12

**Authors:** Shan-You Huang, Chih-Chia Cheng

**Affiliations:** 1 Graduate Institute of Applied Science and Technology, National Taiwan University of Science and Technology, Taipei 10607, Taiwan; dandance901@gmail.com; 2 Advanced Membrane Materials Research Center, National Taiwan University of Science and Technology, Taipei 10607, Taiwan

**Keywords:** amphiphilic interactions, amphiphilic polymers, spontaneous self-assembly, ultrahigh molecular weight, water-soluble single-chain polymeric nanoparticles

## Abstract

Single-chain polymeric nanoparticles (SCPNs) have great potential as functional nanocarriers for drug delivery and bioimaging, but synthetic challenges in terms of final yield and purification procedures limit their use. A new concept to modify and improve the synthetic procedures used to generate water-soluble SCPNs through amphiphilic interactions has been successfully exploited. We developed a new ultrahigh molecular weight amphiphilic polymer containing a hydrophobic poly(epichlorohydrin) backbone and hydrophilic poly(ethylene glycol) side chains. The polymer spontaneously self-assembles into SCPNs in aqueous solution and does not require subsequent purification. The resulting SCPNs possess a number of distinct physical properties, including a uniform hydrodynamic nanoparticle diameter of 10–15 nm, extremely low viscosity and a desirable spherical-like morphology. Concentration-dependent studies demonstrated that stable SCPNs were formed at high concentrations up to 10 mg/mL in aqueous solution, with no significant increase in solution viscosity. Importantly, the SCPNs exhibited high structural stability in media containing serum or phosphate-buffered saline and showed almost no change in hydrodynamic diameter. The combination of these characteristics within a water-soluble SCPN is highly desirable and could potentially be applied in a wide range of biomedical fields. Thus, these findings provide a path towards a new, innovative route for the development of water-soluble SCPNs.

## 1. Introduction

Inspiration from nature has been the principal driving force behind the emergence of new functional polymers with unique and desirable physical characteristics that hold great potential for applications in various fields [1,2]. For example, protein folding is the physical process by which polypeptide chains self-assemble into unique native three-dimensional structures. The conformation of each protein forms in a precise, consistent, reproducible manner to ensure its specific biofunctionality [3]. Protein folding mediated by noncovalent interactions (hydrogen bonding and electrostatic and π–π interactions) [4,5] and hierarchical self-assembly [6] have inspired polymer chemists to synthesize single-chain polymeric nanoparticles (SCPNs) via controlled intramolecular chain folding/collapse of individual polymer chains using physical or chemical crosslinking methods [7,8,9,10,11,12]. These SCPNs exhibit unique physical and chemical properties, including low fluid viscosities, low hydrodynamic volumes and large surface-to-volume ratios [13]. In general, the fabrication of SCPNs relies on folding/collapse and intramolecular crosslinking (either noncovalent complex [14,15,16], dynamic covalent linkage [17,18,19], or covalent bonding [20,21,22]) of a single polymer chain to produce discrete SCPNs. However, when the polymer is present at low concentrations in solution, the presence of active reactive groups/catalyst additives and additional processes (e.g., photoirradiation and heat treatment) are required to induce a conformational change in individual polymer chains from coils to particles via intrachain interactions or chemical linkages. Compared to natural protein folding processes, the fabrication of well-defined SCPNs remains relatively inefficient and ineffective due to the difficulty of promoting and guiding spontaneous formation of SCPNs in solution. Therefore, there is an urgent need to effectively and systematically improve the formation of synthetic SCPNs under different reaction conditions.

Water-soluble SCPNs have attracted significant attention in recent years due to their potential applications in various fields, such as catalysis [23,24,25], drug delivery [26,27,28] and bioimaging [29,30,31]. Current strategies for the synthesis of water-soluble SCPNs are mainly based on noncovalent interactions (intramolecular hydrogen bonds and metal-ligand interactions) [15,23,32,33,34,35] and photochemical reactions [9,21,36] in aqueous solution. However, these synthetic techniques remain limited to the structural design and fabrication of water-soluble SCPNs [9,12,13]. To overcome some limitations in synthetic SCPNs, it is necessary to establish a facile and efficient route for the preparation of water-soluble SCPNs with improved structural stability in aqueous media [37,38]. In our previous work, polyethylene glycol (PEG)-grafted amphiphilic polymers with high grafting densities facilitated polymeric self-assembly in water and efficiently induced phase separation between hydrophilic and hydrophobic segments to promote the formation of water-soluble micelles with a small diameter (<25 nm) [39]. This finding inspired us to further explore a simple and highly efficient route for the synthesis of water-soluble SCPNs. Thus, we boldly conjectured that the introduction of hydrophilic PEG chains into a high-molecular weight hydrophobic polymer backbone may significantly affect the polymer self-assembly process and effectively induce folding/collapse of the polymers into SCPNs in aqueous environments. This new strategy could be expected to greatly improve the formation and stability of SCPNs due to the possible presence of a large volume number of hydrophobic backbones and a combination of hydrophilic and hydrophobic interactions within the SCPN structure [39].

**Scheme 1:**
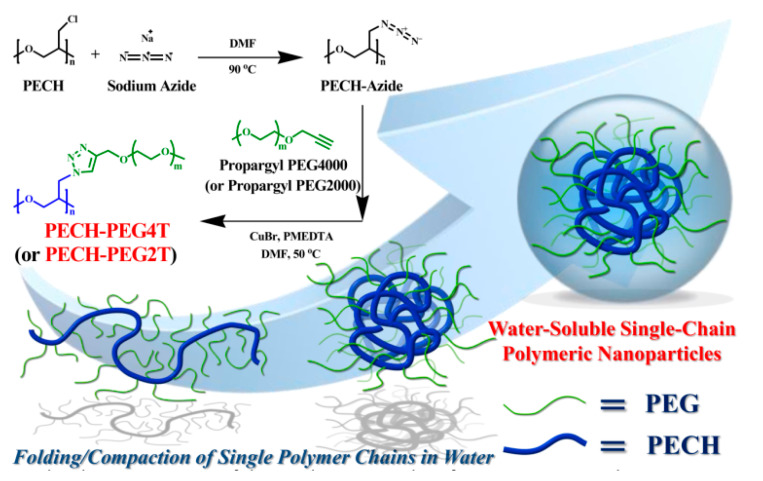
Graphical representation of the synthetic procedures for PECH-PEG4T and PECH-PEG2T via self-assembly of isolated single-chain polymeric nanoparticles (SCPNs) through amphiphilic interactions between PEG-grafted poly(epichlorohydrin (PECH-PEG) polymers.

Herein, we provide a facile route for the preparation of functional amphiphilic polymers that spontaneously self-assemble into SCPNs in water with desirable physical properties. Water-soluble ultrahigh molecular weight amphiphilic polymers containing pendant PEG groups were successfully synthesized via a simple two-step process. The resulting polymers exhibit the excellent property characteristics of SCPNs in water, due to intramolecular chain-folding induced by strong amphiphilic interactions (Scheme 1). We exploited the amphiphilic effects within an ultrahigh molecular weight polymeric coil, whereby a combination of hydrophilicity and hydrophobicity induce folding/collapse of an individual polymer chain into an SCPN with unique physical properties and thus could potentially be used for biomedical applications.

## 2. Materials and Methods

The general materials, material preparations and instrumentation used in this work are described in more detail in the Supplementary Materials.

## 3. Results and Discussion

The new water-soluble amphiphilic polymer, PEG-grafted poly(epichlorohydrin) (PECH-PEG), was prepared through a simple two-step reaction at high yield, as illustrated in Scheme 1. First, poly(epichlorohydrin) with a weight average molecular weight (*M*_w_) of 700,000 g/mol and approximately 7000 repeat units (PECH, Sigma-Aldrich, St Louis, MO, USA) is reacted with sodium azide via a nucleophilic substitution reaction [40]. After purification via flash chromatography, the level of azidation of PECH to azide-grafted PECH (PECH-azide) was calculated by proton and carbon-13 nuclear magnetic resonance spectroscopy (^1^H and ^13^C NMR); complete conversion of chloride to azide was achieved (Figures S1 and S2). In addition, PECH-azide was poorly soluble in water but easily in organic solvents (such as chloroform, tetrahydrofuran and methanol). Subsequently, PECH-azide was further reacted with monopropargyl-terminated PEG [37] via the copper-catalyzed azide-alkyne cycloaddition reaction to achieve the desired PECH-PEG polymers containing pendant PEG chains of different molecular weights (2000 and 4000 g/mol), hereafter termed PECH-PEG2T and PECH-PEG4T, respectively. Both polymers were recovered at high yield (around 70%), displayed the expected chemical structures and possessed extremely high *M*_w_ (higher than ten million g/mol) and broad polydispersity indexes (PDI ≈ 2.0), as determined by ^1^H NMR (Figures S3 and S4) and gel permeation chromatography (GPC; Figure S5 and Table S1), respectively. Although both polymers have a hydrophobic PECH backbone, they are highly soluble in aqueous solutions, even at the high sample concentration of 30 mg/mL. This interesting characteristic prompted us to investigate the influence of the hydrophobic and hydrophilic characteristics of the PECH-PEG polymers on their water solubility.

**Figure 1:**
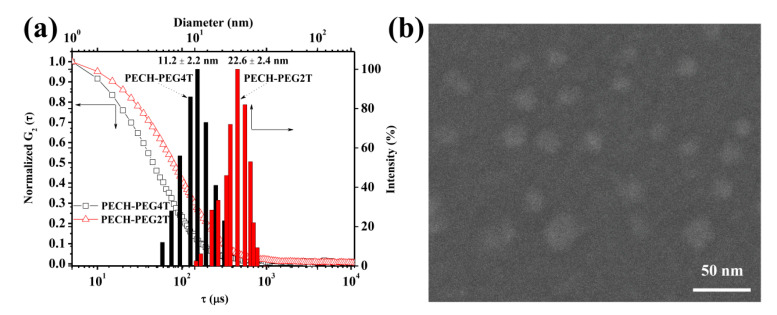
(**a**) Correlation functions and hydrodynamic size distributions of PECH-PEG4T and PECH-PEG2T in water measured at 25 °C. (**b**) SEM image of PECH-PEG4T film spin-coated onto a silicon wafer at 25 °C.

To confirm whether both polymers can self-assemble and form specific nanostructures in aqueous solution, dynamic light scattering (DLS) measurement was performed at 25 °C. Interestingly, DLS analysis of the samples at 1.0 mg/mL in water revealed PECH-PEG4T and PECH-PEG2T had mean hydrodynamic diameters of 11.2 ± 2.2 nm and 22.6 ± 2.4 nm, respectively, (Figure 1a) indicating introduction of the hydrophilic PEG side-chain-induced formation of stable nanosized particles. Moreover, the higher volume fraction of hydrophilic PEG domains in PECH-PEG4T dramatically increased hydrophilicity and prevented intermolecular aggregation of the hydrophobic PECH backbone, leading to the spontaneous generation of nanoparticles with SCPN-like hydrodynamic diameters [7,9,13]. In contrast, the lower hydrophilicity of PECH-PEG2T led to insufficient stability of the PECH backbone in water and resulted in the formation of larger multichain aggregates. To gain deeper insight into the surface morphology and microstructure of the PECH-PEG polymers in water, scanning electron microscopy (SEM) and atomic force microscopy (AFM) were performed at 25 °C. As shown in Figure 1b and Figures S6 and S7, the SEM and AFM images were in almost perfect agreement with the DLS data, and demonstrated that PECH-PEG4T self-assembles into nanoparticles with a small diameter of 10–20 nm. Furthermore, this observation also implies that the robust intramolecular hydrophobic interactions conferred by the high-molecular weight PECH backbone within the interior of the nanoparticles would maintain the stability, particle size and morphology of the self-assembled PECH-PEG4T nanostructures. Based on the above findings, these observations were unexpected because usually, a polymer with high molecular weight has a strong tendency to self-aggregate into large particles in solution; however, the introduction of hydrophilic PEG chains into a high-molecular weight hydrophobic PECH backbone resulted in the formation of nanoparticles with a small diameter (ca.11 nm) in water. Although the exact mechanism is presently unknown, several studies are currently underway to explore the dynamic behavior and molecular simulation of PECH-PEG in aqueous solution.

**Figure 2:**
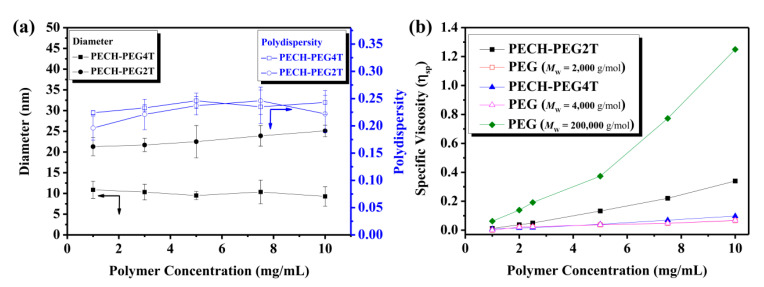
(**a**) Mean hydrodynamic diameter and polydispersity index of various concentrations of PECH-PEG4T and PECH-PEG2T in water. (**b**) Specific viscosity of various concentrations of PECH-PEG4T, PECH-PEG2T and commercial PEG polymers in water.

To further confirm that the amphiphilic nature of the PECH-PECG4T leads to spontaneous formation of structurally stable SCPNs, concentration-dependent DLS and viscosity measurements were conducted for various concentrations (1 to 10 mg/mL) of PECH-PEG4T and PECH-PEG2T in aqueous solution at 25 °C. Surprisingly, DLS (Figure 2a) revealed that the hydrodynamic diameter of PECH-PEG4T remained almost unchanged when the concentration of PECH-PEG4T was increased gradually to 10 mg/mL, whereas the hydrodynamic diameter of PECH-PEG2T gradually increased from 21.3 ± 2.2 to 25.1 ± 1.4 nm. These results indicate that highly stable polymeric SCPNs form independently of the concentration of PECH-PEG4T. Indeed, the presence of sufficient hydrophobic domains within the PECH backbone and an appropriate volume fraction of hydrophilic PEG segments in the structure are likely to substantially improve the stability of spontaneously self-assembled SCPNs in aqueous solution, even at elevated concentrations. In other words, the absence of hydrophilic PEG-rich segments in PECH-PEG2T induced the formation of intermolecular aggregates, leading to a gradual increase in the hydrodynamic diameter as the polymer concentration increased. Next, the viscosities of PECH-PEG4T and PECH-PEG2T in water were determined using a rheometer at 25 °C. As expected, the viscosity data exhibited a similar trend as the concentration-dependent DLS experiment. Figure 2b and Figures S8–S12 show the viscosity of each polymer sample increased linearly with concentration, indicating the fluids were within the dilute solution regime. However, when the concentrations were gradually increased from 1 to 10 mg/mL, PECH-PEG4T solution exhibited almost no increase in viscosity, similarly to the oligomeric PEGs (2000 and 4000 g/mol). This indicates intramolecular hydrophobic interaction-assisted chain folding of the PECH backbone within the polymeric nanoparticles dominates and effectively decreases the affinity for water molecules, which inhibits interchain interactions between polymer chains and prevents an increase in solution viscosity. In contrast, the viscosity of the PECH-PEG2T solution increased gradually with concentration, similarly to high molecular weight PEG (200,000 g/mol). Thus, the SCPNs formed by PECH-PEG4T maintain a spherical-like shape and small size as the polymer concentration increases, due to the presence of a sufficient fraction of hydrophilic PEG segments in PECH-PEG4T, which ensures that the hydrophobic PECH backbone favors intramolecular folding/collapse of polymer chains and formation of isolated SCPNs at high concentrations. In other words, increasing the PEG chain length can dramatically enhance the hydrophilicity of the resulting PECH-PEG4T polymers to induce repulsive interactions between the hydrophilic PEG chains and hydrophobic PECH backbone, while effectively promoting intramolecular collapse of individual polymer chains and suppressing the formation of intermolecular aggregates in water, thus resulting in no significant alteration in solution viscosity.

**Figure 3:**
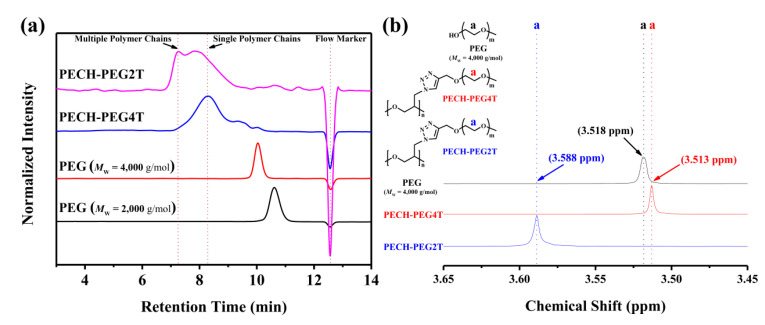
(**a**) Gel permeation chromatography (GPC) traces for PECH-PEG4T, PECH-PEG2T and commercial PEG polymers with water as the eluent at 50 °C. (**b**) ^1^H NMR spectra of PECH-PEG4T, PECH-PEG2T and PEG (*M*_w_ = 4000 g/mol) in D_2_O at 25 °C.

To further explore the effects of intramolecular collapse and intermolecular aggregation in aqueous solution, the molecular weight distributions of PECH-PEG4T and PECH-PEG2T were analyzed using a water-based GPC system at 25 °C. As indicated in Figure 3a, PECH-PEG4T exhibited a significantly smaller hydrodynamic volume and longer retention time in water than PECH-PEG2T, even though PECH-PEG4T has a much higher molecular weight than PECH-PEG2T. This result provides crucial support of our hypothesis, as effective control of amphipathic repulsion between the high-density hydrophilic PEG chains and high-molecular weight hydrophobic PECH backbone successfully induced intramolecular folding/collapse of individual polymer chains into water-soluble SCPNs, and thus hinders intermolecular aggregation and increases structural stability. In addition, PECH-PEG4T had a significantly lower PDI than PECH-PEG2T (Table S2), indicating the formation of self-assembled SCPNs with a relatively ordered arrangement. In order to verify whether the PECH backbone significantly affects the structural features of PEG segments in water, ^1^H NMR spectra were measured for 10 mg/mL solutions of oligomeric PEG, PECH-PEG2T and PECH-PEG4T in D_2_O at 25 °C. Figure 3b clearly indicates that the proton peak of the PEG segments of PECH-PEG4T shifted slightly upfield and was narrower than those of oligomeric PEG, whereas the proton peaks for PECH-PEG2T shifted substantially downfield and became broader. This suggests that the upfield chemical shift for PECH-PEG4T can be attributed to hydrophobic interactions between the PECH backbone and the sufficient lengths and amounts of hydrophilic PEG chains, resulting in freer and more fully chain-extended PEG segments in aqueous medium. In contrast, due to insufficient hydrophilicity to maintain structural integrity, PECH-PEG2T rapidly undergoes intermolecular self-assembly in water to form multichain nanoaggregates that are larger than SCPNs, which cause a substantial downfield shift and peak shape changes. Overall, these observations confirm that amphiphilic PECH-PEG4T spontaneously self-assembles to form highly stable SCPNs in aqueous solution.

Next, we further characterized the structural stability of SCPNs in phosphate-buffered saline (PBS) and Dulbecco’s modified eagle medium (DMEM) containing 10% fetal bovine serum (FBS), which act as strong nanoparticle-destabilizing environments under physiological conditions. As shown in Figures S13 and S14, the hydrodynamic diameter of PECH-PEG4T did not significantly change after 24 h at 37 °C in either media, indicating high structural stability in biological media due to the appropriate balance between the hydrophobic and hydrophilic interactions within the SCPNs, indicating the SCPNs have potential for biomedical applications. Collectively, these findings clearly demonstrate that the high-molecular weight hydrophobic PECH backbone and sufficiently long PEG chains within the polymeric structure induce hydrophobic and hydrophilic interactions and facilitate folding of the single-chain PECH backbone. Thus, PECH-PEG4T spontaneously self-assembles into isolated SCPNs in aqueous solution with unique physical characteristics and excellent structural stability. To the best of our knowledge, there are no reports of other ultrahigh molecular weight water-soluble amphiphilic polymers that spontaneously form SCPNs with high structural stability, in the absence of active reactive groups/catalyst additives or that do not require additional processes. Thus, PECH-PEG4T could serve as a rapid and efficient synthetic route for the development of water-soluble SCPNs.

## 4. Conclusions

In summary, we successfully confirmed a facile, highly efficient synthetic route for development of water-soluble SCPNs based on the presence of a high-molecular weight hydrophobic PECH backbone and hydrophilic pendant PEG chains. Due to the presence of sufficient hydrophobic domains within the PECH backbone and an appropriate volume fraction of hydrophilic PEG segments in the polymer structure, PECH-PEG4T spontaneously self-assembles into well-defined, stable SCPNs in water with distinct physical properties, including a small hydrodynamic diameter of 10–15 nm, extremely low viscosity and a desirable spherical-like morphology. Concentration-dependent experiments indicated that effective control of amphiphilic repulsion between the high-density hydrophilic PEG chains and high-molecular weight hydrophobic PECH backbone induces efficient intramolecular folding/collapse of individual polymer chains into water-soluble SCPNs, even at high concentrations in aqueous solution. Moreover, the resulting SCPNs exhibit high structural stability in PBS and DMEM containing FBS, showing almost no change in hydrodynamic diameter over 24 h, suggesting PECH-PEG4T could potentially be applied in a wide range of biomedical fields. Thus, this study offers a facile and effective route towards the synthesis of water-soluble SCPNs that could potentially help with the development of existing single-chain technology.

## Supplementary Materials

The following are available online at https://www.mdpi.com/2079-4991/10/10/2006/s1, Figure S1: 1H NMR spectra of PECH and PECH-azide in deuterated chloroform (CDCl3) at 25 °C, Figure S2: 13C NMR spectra of PECH and PECH-azide in CDCl3 at 25 °C, Figure S3: 1H NMR spectrum of PECH-PEG2T in CDCl3 at 25 °C, Figure S4: 1H NMR spectrum of PECH-PEG4T in CDCl3 at 25 °C, Figure S5: GPC traces for PEG (MW = 4000 g/mol), PECH-azide, PECH-PEG2T and PECH-PEG4T with DMF as the eluent at 50 °C, Figure S6: AFM image of spin-coated PECH-PEG4T film on silicon wafer at 25 °C, Figure S7: AFM image of spin-coated PECH-PEG2T film on silicon wafer at 25 °C, Figure S8: Viscosity as a function of shear rate for aqueous solutions containing various concentrations of PEG (MW = 2000 g/mol) at 25 °C, Figure S9: Viscosity as a function of shear rate for aqueous solutions containing various concentrations of PEG (MW = 4000 g/mol) at 25 °C, Figure S10: Viscosity as a function of shear rate for aqueous solutions containing various concentrations of PEG (MW = 200,000 g/mol) at 25 °C, Figure S11: Viscosity as a function of shear rate for aqueous solutions containing various concentrations of PECH-PEG2T at 25 °C, Figure S12: Viscosity as a function of shear rate for aqueous solutions containing various concentrations of PECH-PEG4T at 25 °C, Figure S13: DLS analysis of PECH-PEG4T after incubation in various media for 24 h at 37 °C, Figure S14: Kinetic stability of PECH-PEG2T and PECH-PEG4T nanoparticles in PBS at pH 7.4 and 37 °C over time, Table S1: GPC analysis of PECH and derivatives in DMF as the mobile phase at a flow rate of 1.0 mL/min, Table S2: GPC analysis of PECH and derivatives in water as the mobile phase at a flow rate of 1.0 mL/min.
