# Supplementary material for: Spontaneous Self-Assembly of Single-Chain Amphiphilic Polymeric Nanoparticles in Water
Source: Nanomaterials (Basel). 2020 Oct 12;10(10):2006. doi: 10.3390/nano10102006 (PMC7601091; doi:10.3390/nano10102006)
Supplement: Supplementary file 1 [file nanomaterials-10-02006-s001.pdf]

Supplementary Materials

Spontaneous Self-Assembly of Single-Chain Amphiphilic Polymeric Nanoparticles in Water

Shan-You Huang[1] and Chih-Chia Cheng[1,2]*

1. Graduate Institute of Applied Science and Technology, National Taiwan University of Science and Technology, Taipei 10607, Taiwan. E-mail: cccheng@mail.ntust.edu.tw
2. Advanced Membrane Materials Research Center, National Taiwan University of Science and Technology, Taipei 10607, Taiwan.

* Corresponding Author

Experimental Section

Materials

Polyepichlorohydrin (PECH; M_w = 700,000 g/mol) and polyethylene glycol monomethyl ether (PEG, M_w = 2,000 and 4,000 g/mol) were purchased from Sigma-Aldrich (St. Louis, MO, USA). Scheme 1 shows the chemical structures of the commercial polymers used in this work. All chemicals, reagents and organic solvents were of analytical grade, purchased from Sigma-Aldrich, Showa (Tokyo, Japan) or TEDIA (Fairfield, OH, USA) and used directly without further purification. Phosphate-buffered saline (PBS), Dulbecco's modified eagle's medium (DMEM) and fetal bovine serum (FBS) were obtained from Thermo Fisher Scientific (Waltham, MA, USA) for the long-term structural stability experiments.

Characterization

Proton Nuclear Magnetic Resonance (^{1}H NMR). ^{1}H NMR spectra were obtained using Bruker AVIII 500 MHz spectrometers (Billerica, MA, USA). Typically, NMR samples containing 0.8 mL of deuterated solvent and 10-20 mg sample were analyzed at 25 ℃. *Gel Permeation Chromatography* (GPC). Average molecular weights and the polydispersity indexes were measured on a Waters 1515 HPLC system (Milford, MA, USA) with dimethylformamide (DMF) or water as the mobile phases on Waters Styragel® columns (HR1, HR2, HR3, and HR4). The flow rate and temperature of the mobile phases were 1.0 mL/min and 50 ℃. A molecular weight calibration curve was produced using commercial narrow molecular weight distribution polystyrene or PEG standards (Polymer Standards Service, Silver Spring, MD, USA). *Dynamic Light Scattering (DLS) analysis.* Particle size distributions and hydrodynamic diameters of aqueous solutions were determined using a Nano Brook 90 Plus PALS instrument (Brookhaven Instruments Corp., Holtsville, NY, USA). Sample solutions were incubated at 25 ℃ or 37 ℃ for at least 1 h prior to measurements. *Scanning Electron Microscopy (SEM) and Atomic Force Microscopy (AFM).* The microstructure and surface morphology of polymers were assessed using a field-emission SEM (JSM-6500F, JEOL, Tokyo, Japan) and tapping-mode AFM (NX10, AFM Park Systems, Suwon, South Korea). Sample solutions were spin-coated onto silicon wafer substrates at 1000 rpm for 30 s using a spin coater (SC-80R+S, Yotec Instruments CO., Ltd., Hsinchu City, Taiwan) and dried at room temperature under ambient pressure. *Viscosity Measurements.* Specific viscosity was determined from dynamic viscosity measurements obtained using a MCR 302 rheometer (Anton Paar, Gratz, Austria), as previously described [S1]. **Figure 2b** shows the specific

viscosity values of polymer solutions obtained after applying shear rates between 10 and 100/s (**Figures S8-S12**).

Syntheses of PECH-PEG2T and PECH-PEG4T polymers

Monopropargyl-terminated PEG (propargyl PEG4000 or propargyl PEG2000) and azide-functionalized PECH (PECH-Azide) were synthesized as previously described [S2-S4]. Monopropargyl-terminated PEG (10 g for propargyl PEG4000 or 5 g for propargyl PEG2000, 2.5 mmol) and PECH-Azide (0.1 g, 0.143 μmol) were dissolved in 100 mL of anhydrous DMF at room temperature. Copper (I) bromide (CuBr, 0.009 g, 0.06 mmol) and 1,1,4,7,7-pentamethyldiethylenetriamine (PMDETA; 5.9 μL, 0.028 mmol) were sequentially added and the reaction mixture was heated to 50 ℃ until the disappearance of the azide peak (at 2095 cm^{-1}) from the Fourier transform infrared (FTIR) spectrum. After evaporating the solvent via vacuum distillation, the residue was subjected to flash column chromatography on neutral aluminum oxide using tetrahydrofuran as the eluent to remove the CuBr and PMDETA catalyst, then purified by dialysis against methanol using a cellulose membrane (molecular weight cut off, 25,000 g/mol) for 3 days. Finally, the methanol solvent was removed by rotary evaporation and dried in a vacuum oven at 40 ℃ to give PECH-PEG2T (1.4 g, yield = 72%) or PECH-PEG4T (2.5 g, yield = 67%).

Preparations of PECH-PEG2T and PECH-PEG4T Nanoparticles

PECH-PEG2T (or PECH-PEG4T) polymer was dissolved in water, followed by continuous sonication for 10 min at 50 ℃, then the solution was cooled to 25 ℃ and used to characterize its physical properties.

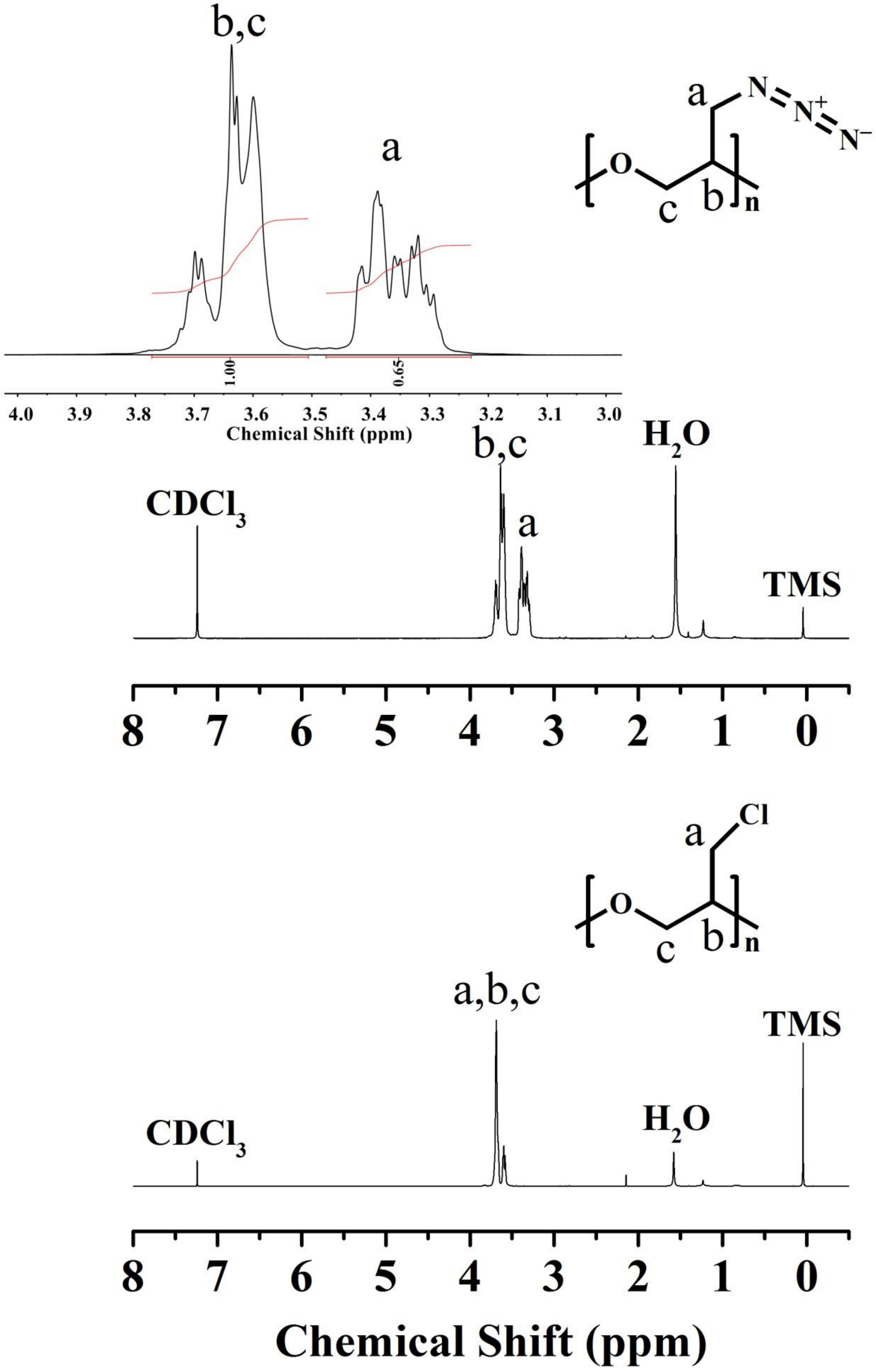

Figure S1: ^{1}H NMR spectra of PECH and PECH-Azide in deuterated chloroform ($CDCl_3$) at 25 ℃.

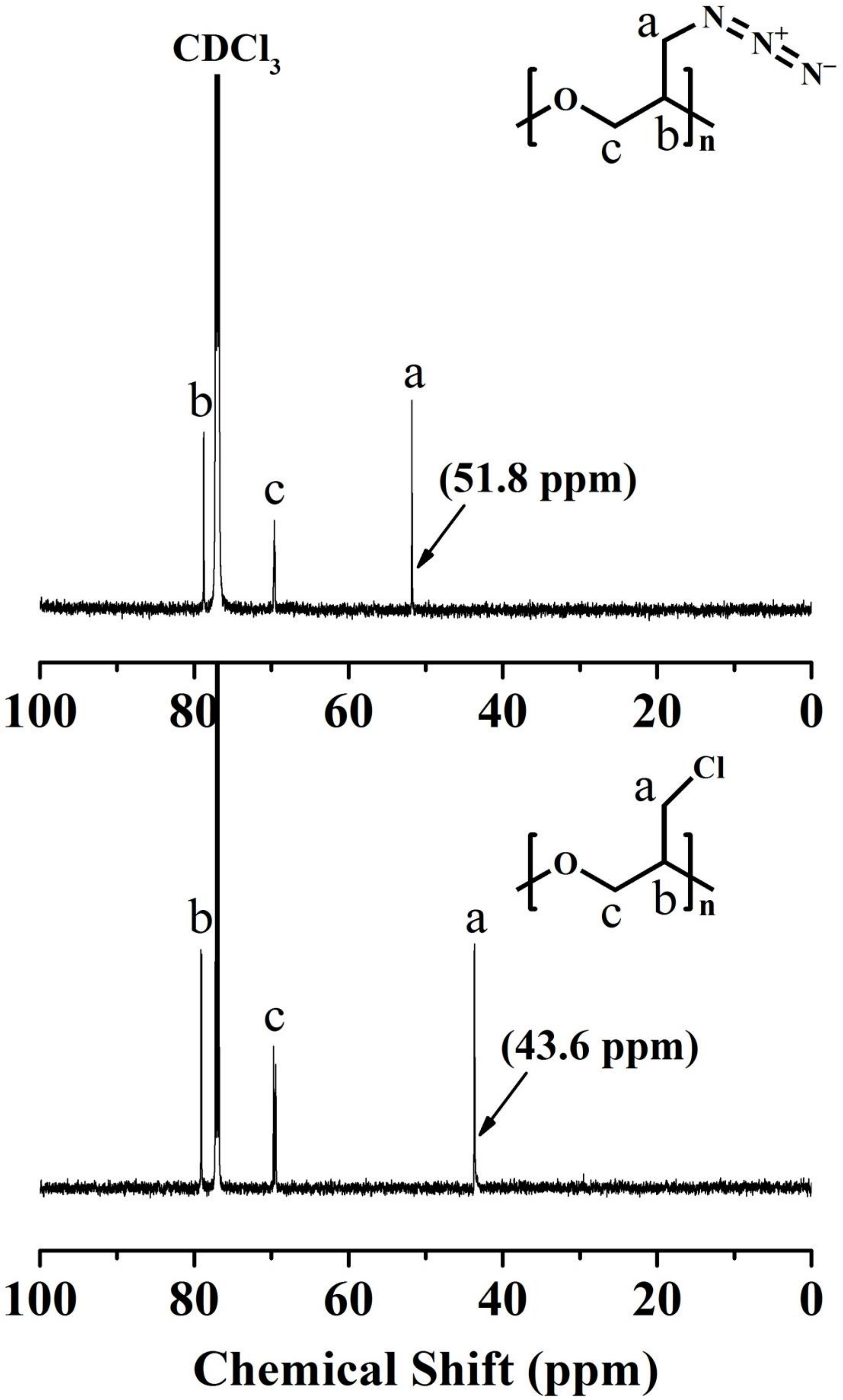

Figure S2: ^{13}C NMR spectra of PECH and PECH-Azide in $CDCl_3$ at 25 ℃.

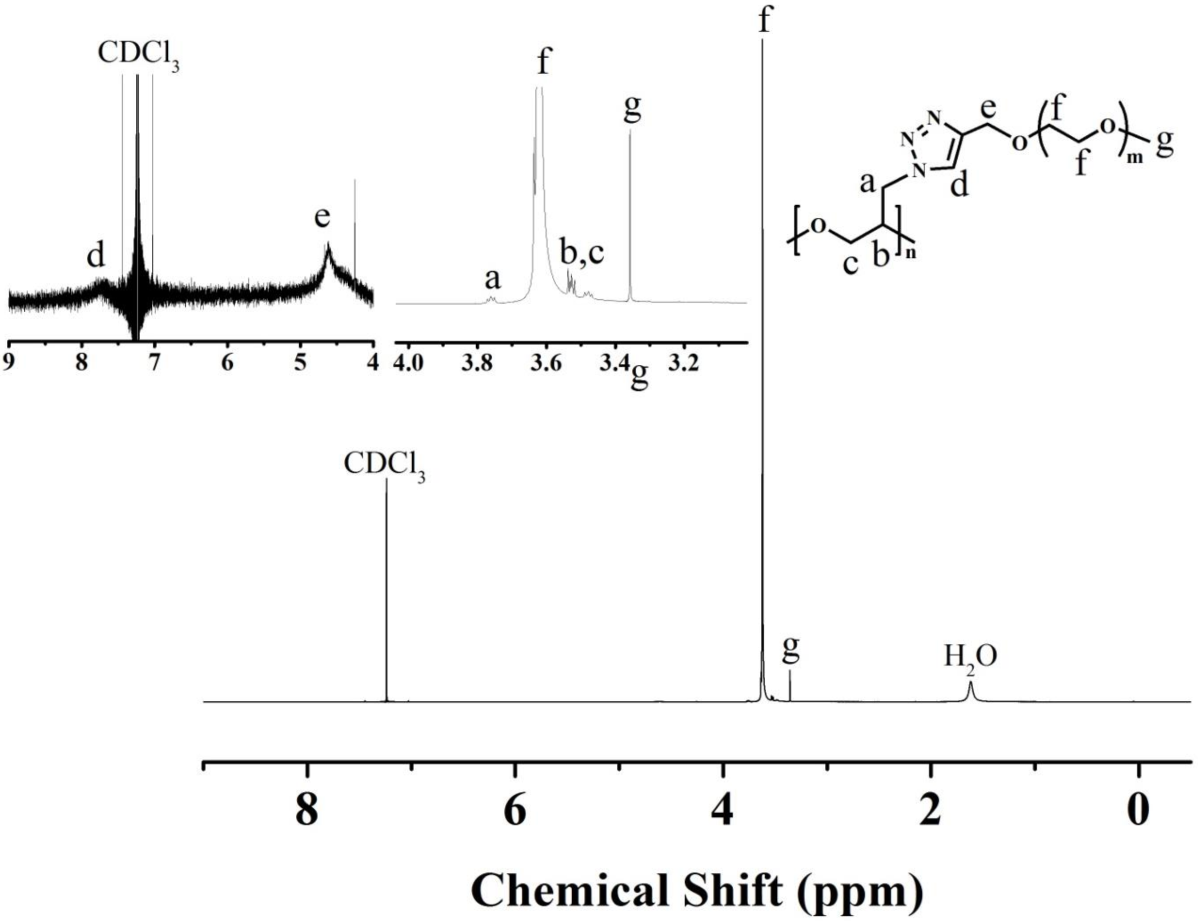

Figure S3: ^{1}H NMR spectrum of PECH-PEG2T in $CDCl_3$ at 25 ℃.

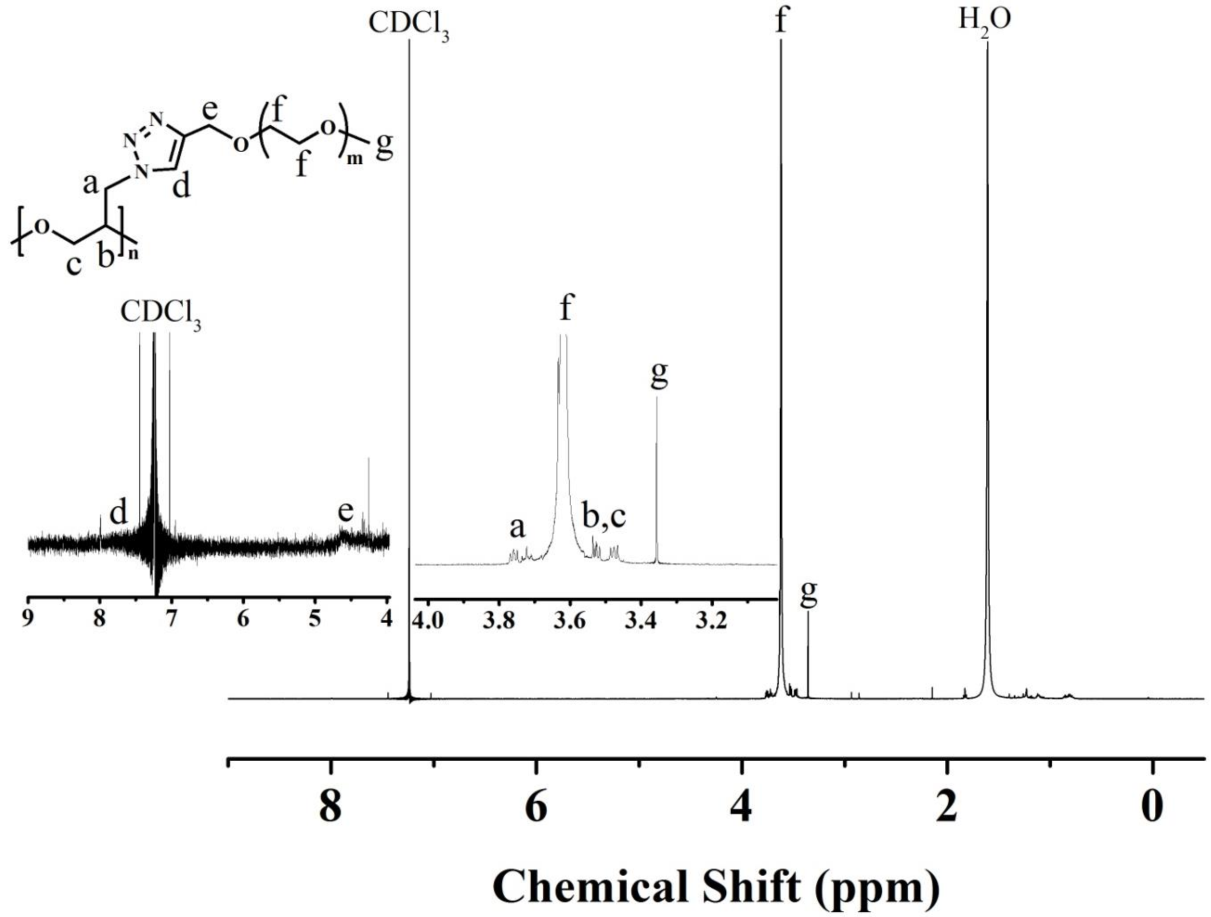

Figure S4: ^{1}H NMR spectrum of PECH-PEG4T in $CDCl_3$ at 25 ℃.

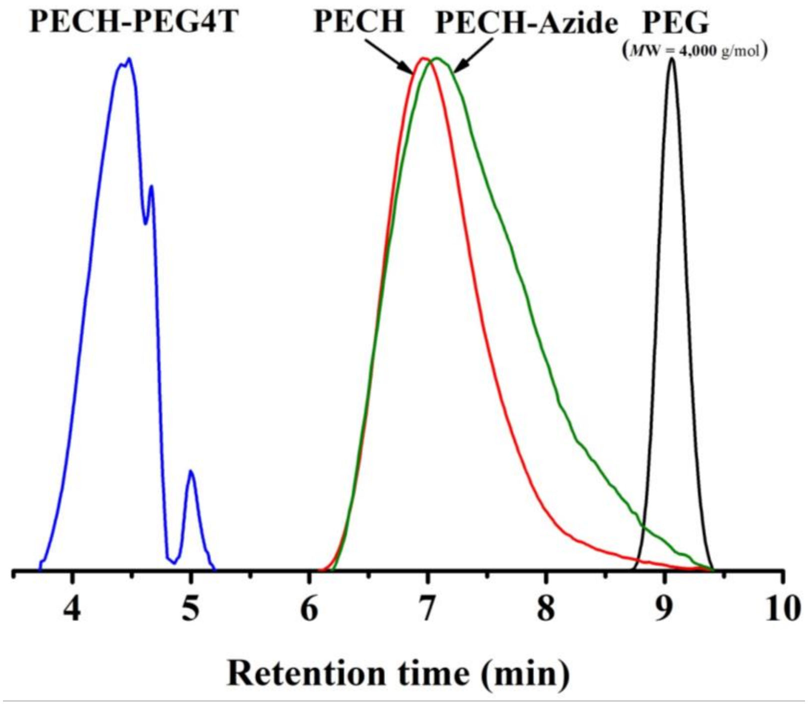

Figure S5: GPC traces for PEG (M_W = 4,000 g/mol), PECH-Azide, PECH-PEG2T and PECH-PEG4T with DMF as the eluent at 50 ℃.

Table S1: GPC analysis of PECH and derivatives in DMF as the mobile phase at a flow rate of 1.0 mL/min.

Sample	M_w[a]	M_n[b]	PDI[c]
PEG (M_w = 2,000 g/mol)	2,100	2,040	1.03
PEG (M_w = 4,000 g/mol)	4,400	4.310	1.02
PECH	68,200	42,600	1.60
PECH-Azide	64,500	35,400	1.82
PECH-PEG2T	49,766,000	26,192,000	1.90
PECH-PEG4T	64,633,000	34,563,000	1.87

a. M_w = weight average molecular weight.

b. M_n = number average molecular weight.

c. PDI = polydispersity index.

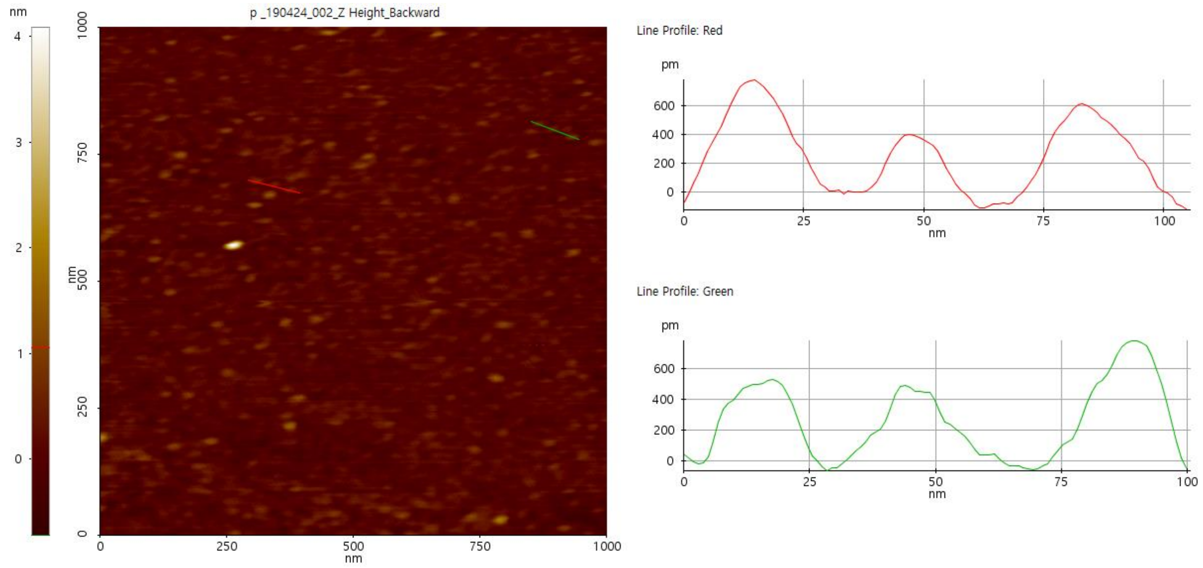

Figure. S6: AFM image of spin-coated PECH-PEG4T film on silicon wafer at 25 ℃.

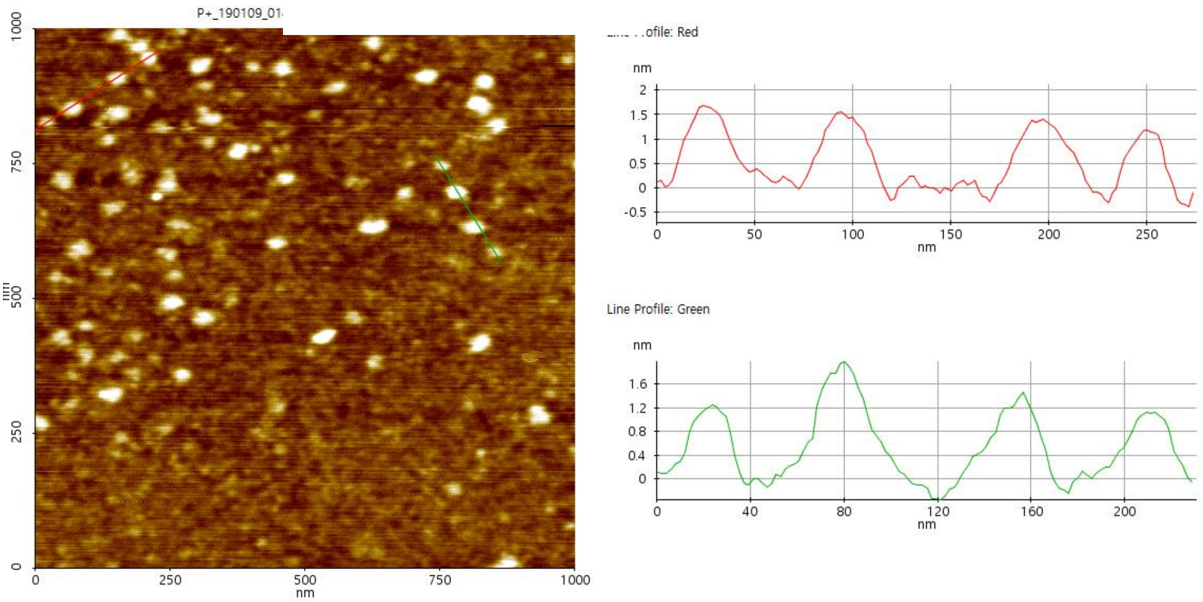

Figure. S7: AFM image of spin-coated PECH-PEG2T film on silicon wafer at 25 ℃.

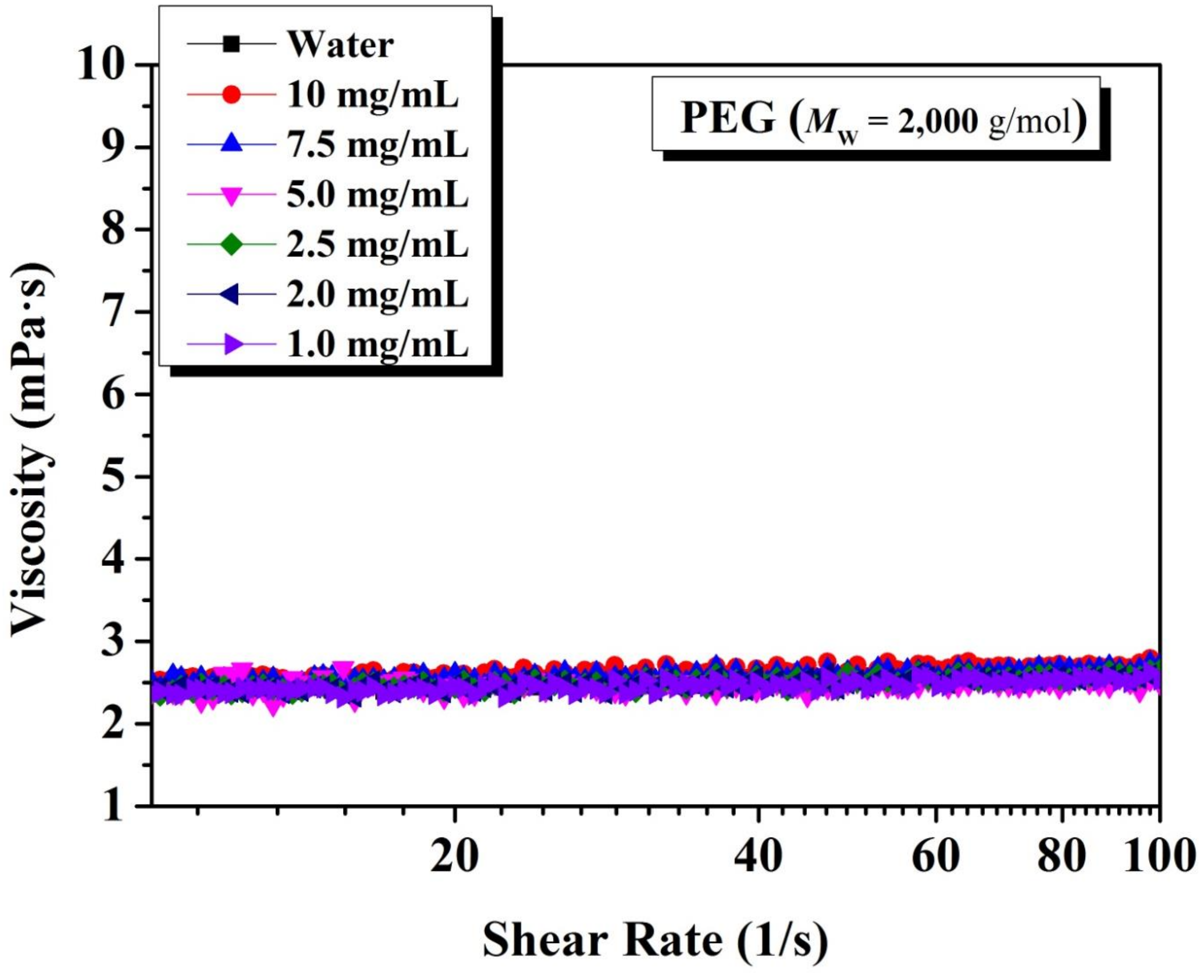

Figure S8: Viscosity as a function of shear rate for aqueous solutions containing various concentrations of PEG (M_W = 2,000 g/mol) at 25 ℃.

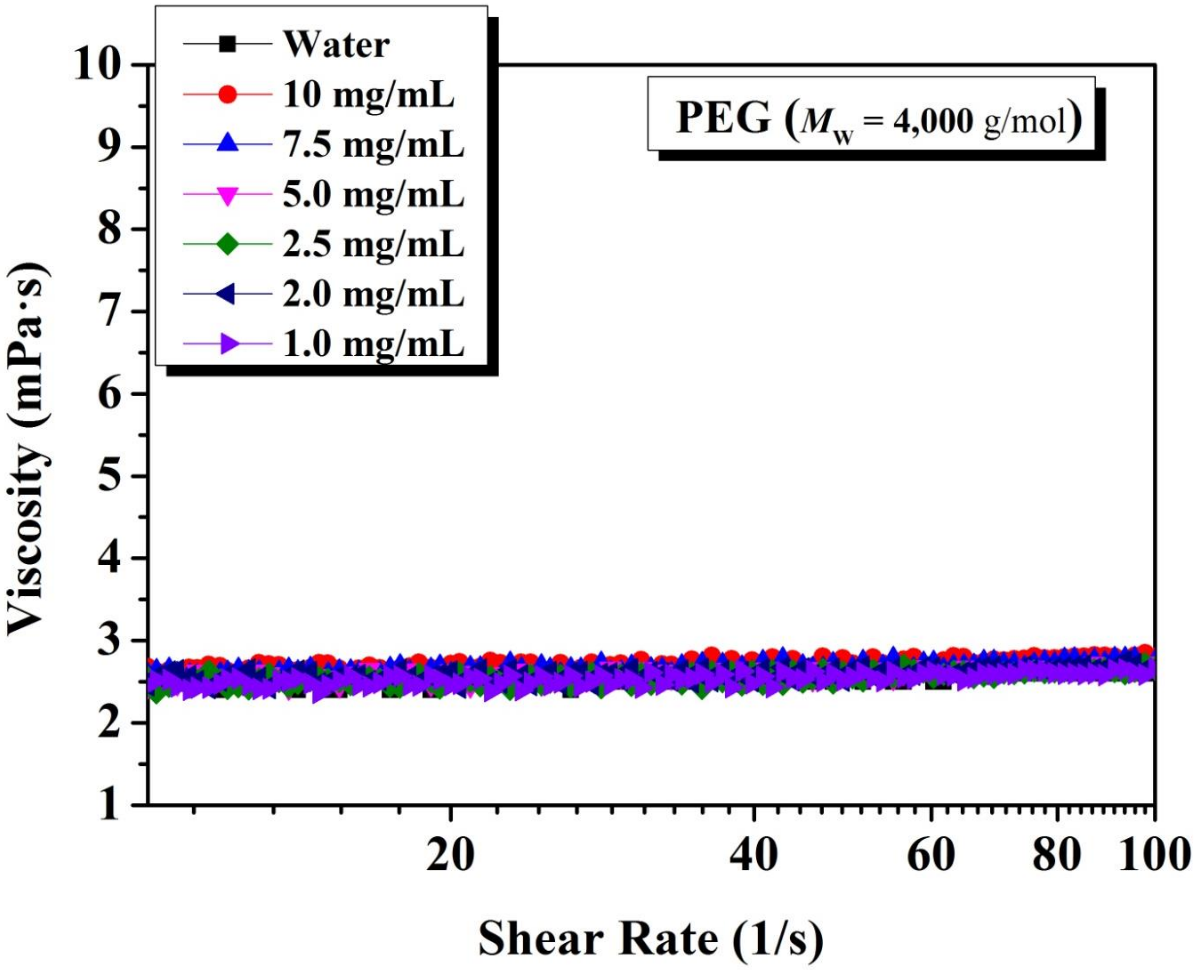

Figure S9: Viscosity as a function of shear rate for aqueous solutions containing various concentrations of PEG (M_W = 4,000 g/mol) at 25 ℃.

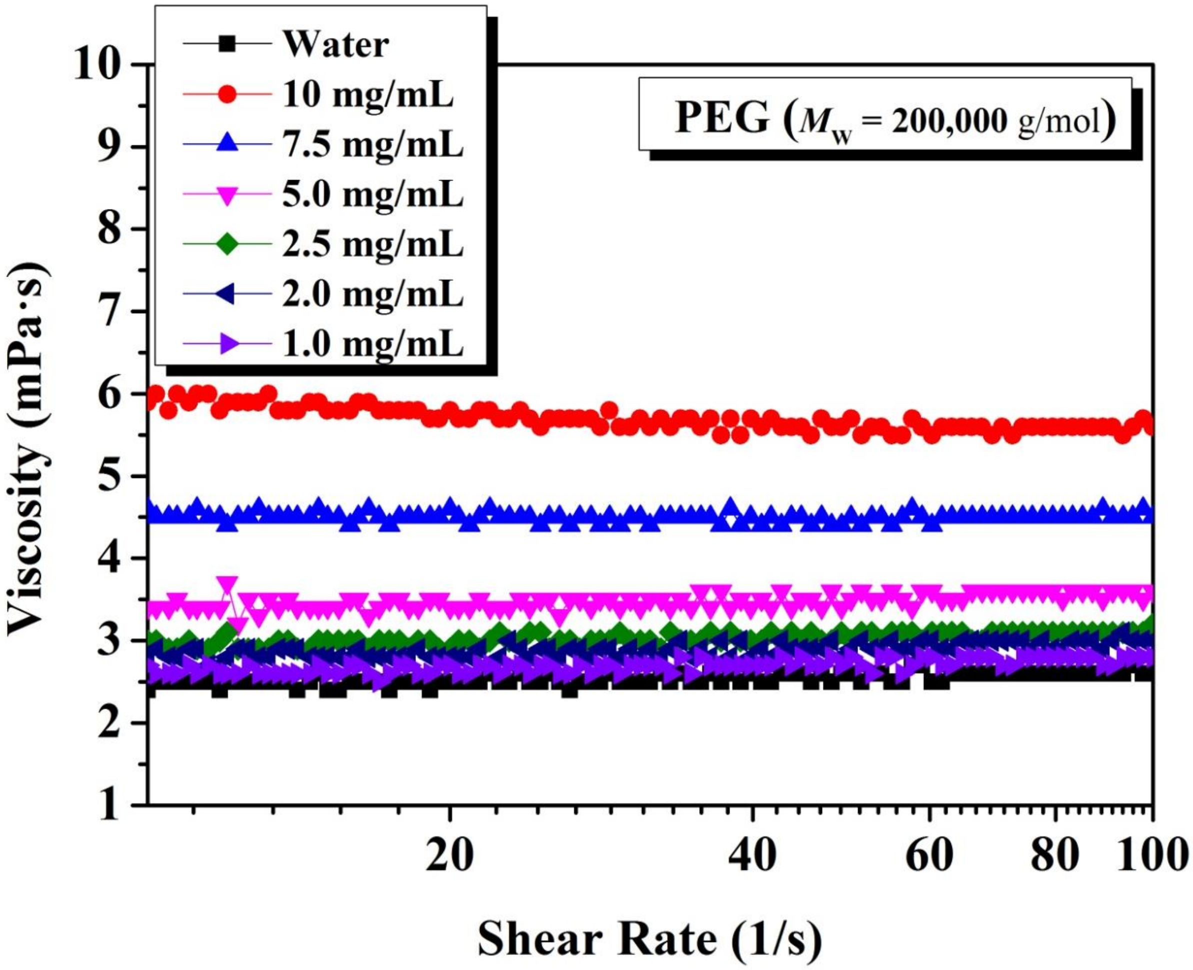

Figure S10: Viscosity as a function of shear rate for aqueous solutions containing various concentrations of PEG (M_W = 200,000 g/mol) at 25 ℃.

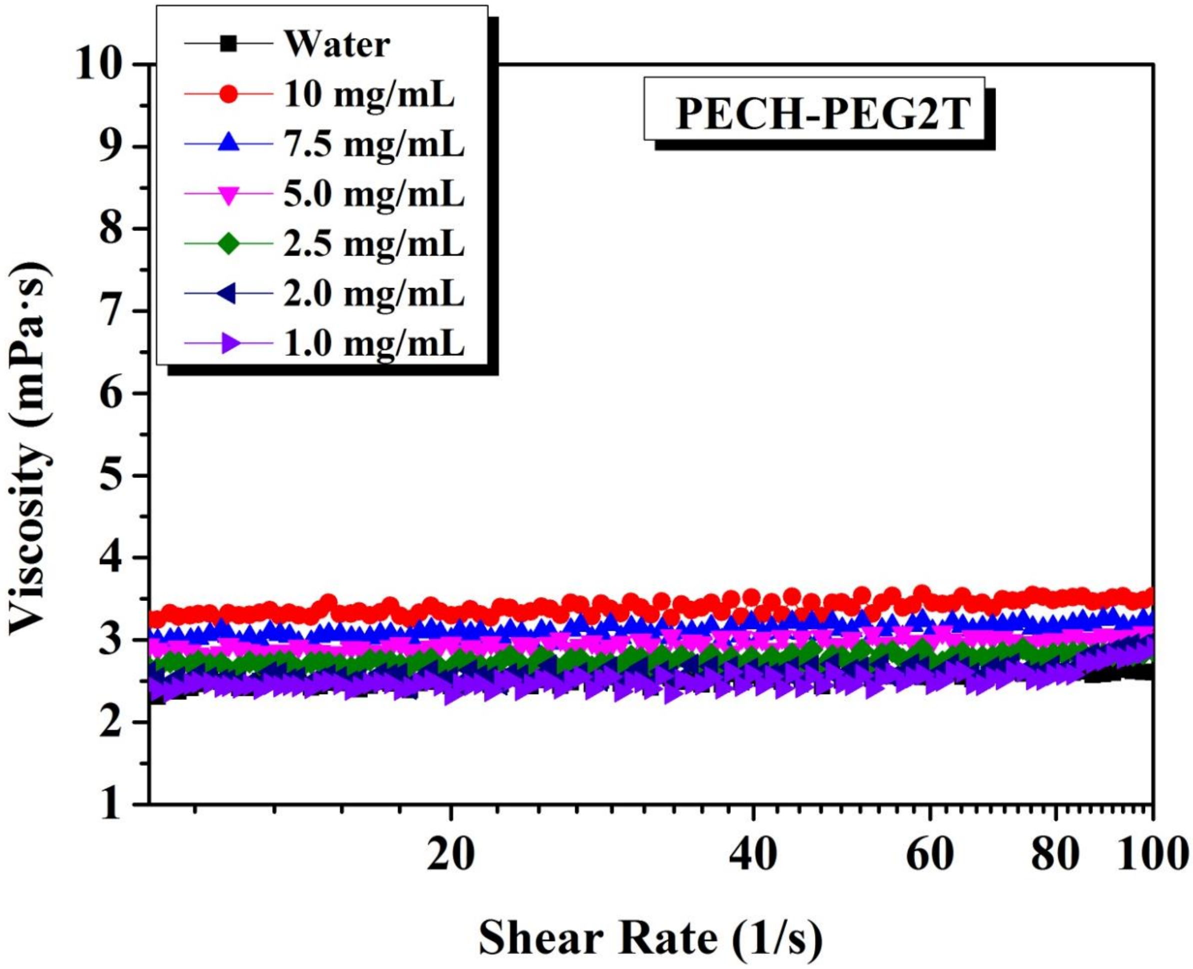

Figure S11: Viscosity as a function of shear rate for aqueous solutions containing various concentrations of PECH-PEG2T at 25 ℃.

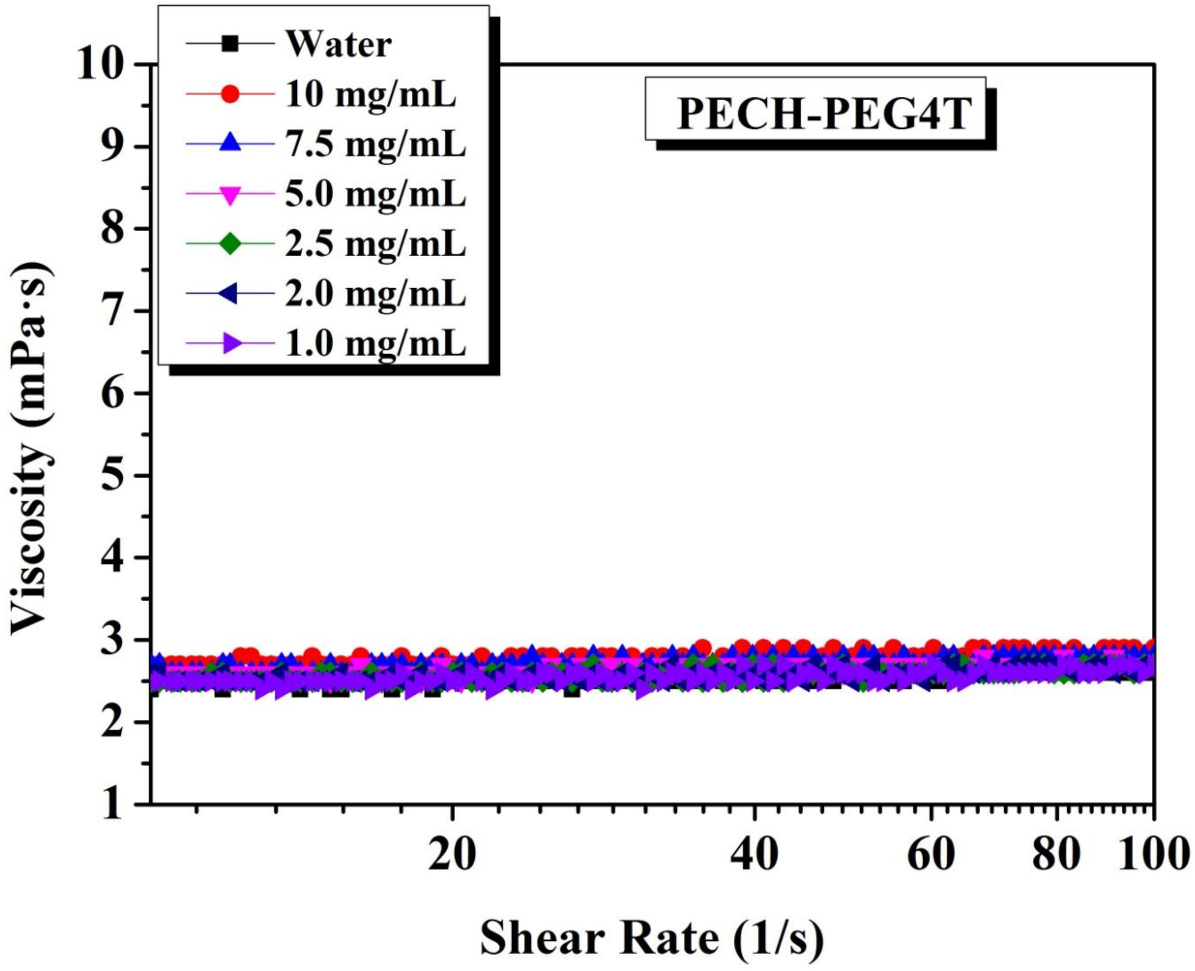

Figure S12: Viscosity as a function of shear rate for aqueous solutions containing various concentrations of PECH-PEG4T at 25 ℃.

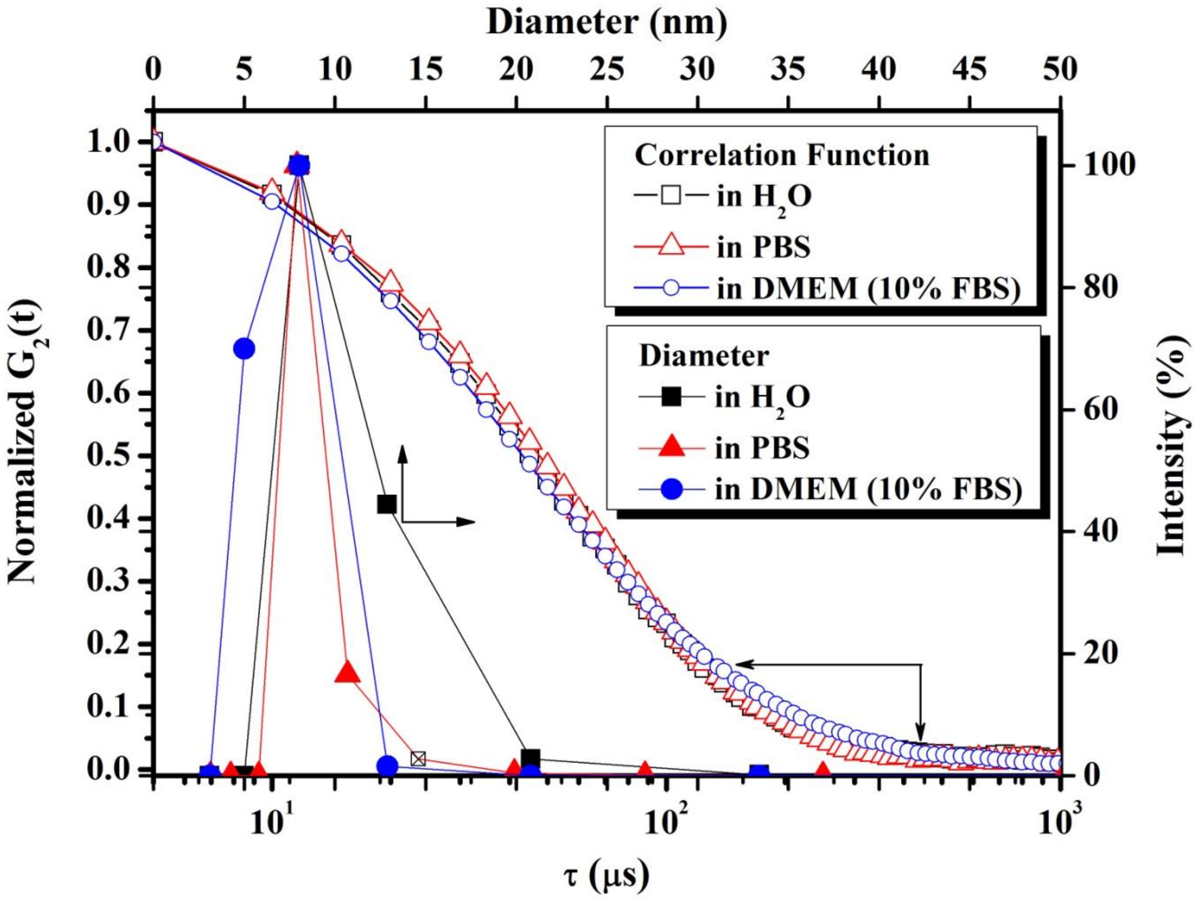

Figure S13: DLS analysis of PECH-PEG4T after incubation in various media for 24 h at 37 ℃.

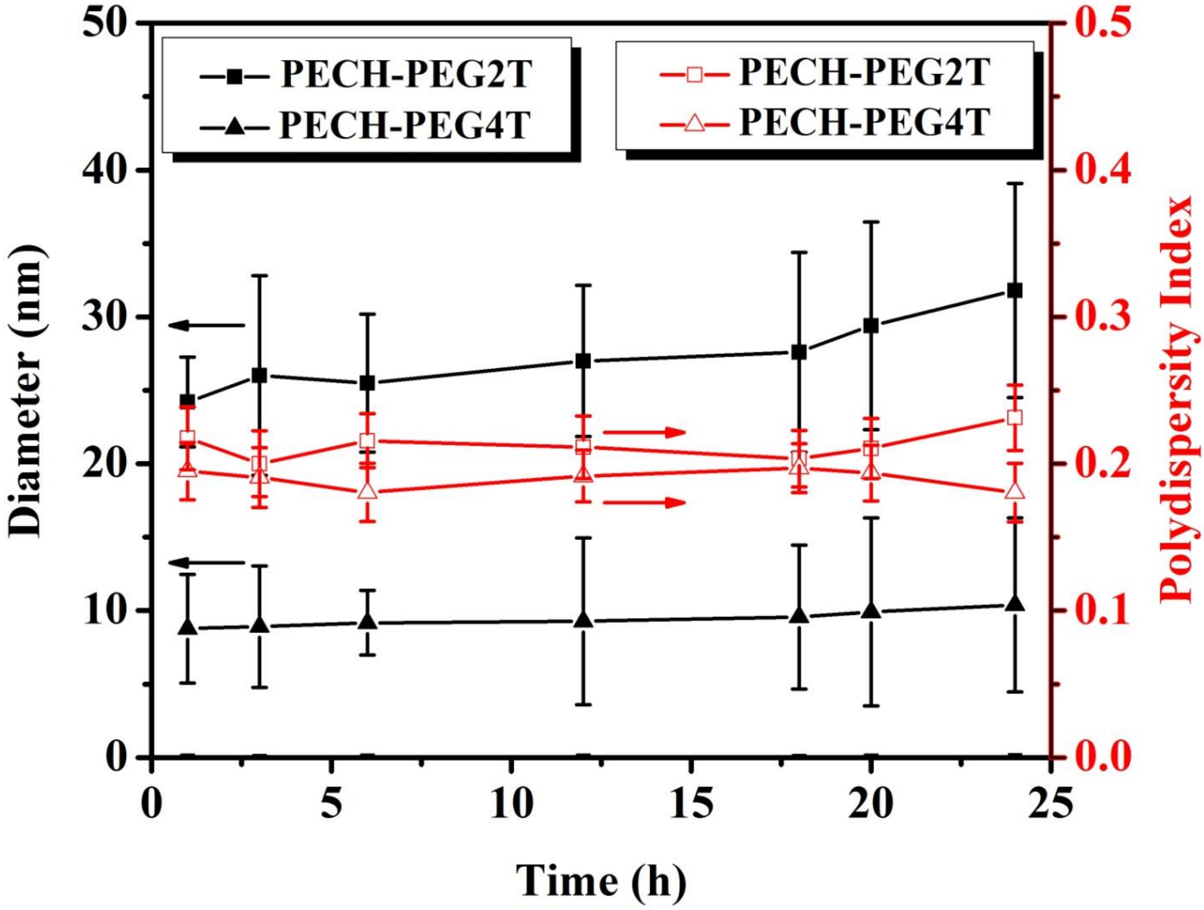

Figure S14: Kinetic stability of PECH-PEG2T and PECH-PEG4T nanoparticles in PBS at pH 7.4 and 37 ℃ over time.

After 24 h of monitoring, the hydrodynamic diameter and polydispersity index of PECH-PEG4T in aqueous solution remained almost unchanged, indicating high structural stability in PBS due to the appropriate balance between the hydrophobic and hydrophilic interactions within the SCPNs. Conversely, the hydrodynamic diameter of PECH-PEG2T nanoparticles showed an almost linear increase over time, suggesting that PECH-PEG2T nanoparticles have a high tendency to form aggregates due to insufficient hydrophilicity to maintain structural integrity.

Table S2: GPC analysis of PECH and derivatives in water as the mobile phase at a flow rate of 1.0 mL/min.

Sample	M_w[a]	M_n[b]	PDI[c]
PEG (M_w = 2,000 g/mol)	670	560	1.20
PEG (M_w = 4,000 g/mol)	1680	1560	1.07
PECH-PEG2T	110500	55000	2.01
PECH-PEG4T	88200	53100	1.66

a. M_w = weight average molecular weight.

b. M_n = number average molecular weight.

c. PDI = polydispersity index.

References:

S1. Galant, O.; Davidovich-Pinhas, M.; Diesendruck, C.E. The effect of intramolecular cross-linking on polymer interactions in solution. *Macromol. Rapid Commun.* **2018**, *39*, 1800407.

S2. Binder, W.H.; Sachsenhofer, R. Polymersome/silica capsules by ‘click’-chemistry. *Macromol. Rapid Commun.* **2008**, *29*, 1097–1103.

S3. Liao, Z.S.; Huang, S.Y.; Huang, J.J.; Chen, J.K.; Lee, A.W.; Lai, J.Y.; Lee, D.J.; Cheng, C.C. Self-assembled pH-responsive polymeric micelles for highly efficient, non-cytotoxic delivery of doxorubicin chemotherapy to inhibit macrophage activation: *in vitro* investigation. *Biomacromolecules* **2018**, *19*, 2772–2781.

S4. Brochu, S.; Ampleman, G. Synthesis and characterization of glycidyl azide polymers using isotactic and chiral poly(epichlorohydrin)s. *Macromolecules* **1996**, *29*, 5539–5545.
